# Saccharification of recalcitrant biomass and integration options for lignocellulosic sugars from Catchlight Energy’s sugar process (CLE Sugar)

**DOI:** 10.1186/1754-6834-6-10

**Published:** 2013-01-28

**Authors:** Johnway Gao, Dwight Anderson, Benjamin Levie

**Affiliations:** 1 Catchlight Energy LLC, 98063, Federal Way, WA, USA

**Keywords:** CLE Sugar, Mild bisulfite pretreatment, Recalcitrant, Softwood, Loblolly pine, Hardwood, Maple, Switchgrass, Lignocellulosic sugar, Syrup, Sugar solid, Enzyme, Hydrolysis, Biofuel, Ethanol

## Abstract

**Background:**

Woody biomass is one of the most abundant biomass feedstocks, besides agriculture residuals in the United States. The sustainable harvest residuals and thinnings alone are estimated at about 75 million tons/year. These forest residuals and thinnings could produce the equivalent of 5 billion gallons of lignocellulosic ethanol annually. Softwood biomass is the most recalcitrant biomass in pretreatment before an enzymatic hydrolysis. To utilize the most recalcitrant lignocellulosic materials, an efficient, industrially scalable and cost effective pretreatment method is needed.

**Results:**

Obtaining a high yield of sugar from recalcitrant biomass generally requires a high severity of pretreatment with aggressive chemistry, followed by extensive conditioning, and large doses of enzymes. Catchlight Energy’s Sugar process, CLE Sugar, uses a low intensity, high throughput variation of bisulfite pulping to pretreat recalcitrant biomass, such as softwood forest residuals. By leveraging well-proven bisulfite technology and the rapid progress of enzyme suppliers, CLE Sugar can achieve a high yield of total biomass carbohydrate conversion to monomeric lignocellulosic sugars. For example, 85.8% of biomass carbohydrates are saccharified for un-debarked Loblolly pine chips (softwood), and 94.0% for debarked maple chips (hardwood). Furan compound formation was 1.29% of biomass feedstock for Loblolly pine and 1.10% for maple. At 17% solids hydrolysis of pretreated softwood, an enzyme dose of 0.075 g Sigma enzyme mixture/g dry pretreated (unwashed) biomass was needed to achieve 8.1% total sugar titer in the hydrolysate and an overall prehydrolysate liquor plus enzymatic hydrolysis conversion yield of 76.6%. At a much lower enzyme dosage of 0.044 g CTec2 enzyme product/g dry (unwashed) pretreated softwood, hydrolysis at 17% solids achieved 9.2% total sugar titer in the hydrolysate with an overall sugar yield of 85.0% in the combined prehydrolysate liquor and enzymatic hydrolysate. CLE Sugar has been demonstrated to be effective on hardwood and herbaceous biomass, making it truly feedstock flexible.

**Conclusions:**

Different options exist for integrating lignocellulosic sugar into sugar-using operations. A sugar conversion plant may be adjacent to a CLE Sugar plant, and the CLE Sugar can be concentrated from the initial 10% sugar as needed. Concentrated sugars, however, can be shipped to remote sites such as ethanol plants or other sugar users. In such cases, options for shipping a dense form of sugars include (1) pretreated biomass with enzyme addition, (2) lignocellulosic sugar syrup, and (3) lignocellulosic sugar solid. These could provide the advantage of maximizing the use of existing assets.

## Background

Woody biomass is one of the most abundant biomass feedstocks, besides agriculture residuals in the United States. The sustainable harvest residuals and thinnings alone are estimated at about 75 million tons/year accordingly to the DOE biomass report [1]. These forest residuals and thinnings could produce the equivalent of 5 billion gallons of lignocellulosic ethanol annually.

Woody biomass feedstocks include softwood and hardwood. Various pretreatment methods have been developed for pretreating biomass before an enzymatic conversion of pretreated biomass into monomeric sugars for a biofuel or a bioproduct conversion process. An overview on lignocellulosic biomass pretreatment methods well described that most of the pretreating methods worked well on herbaceous biomass and hardwood biomass while not working well with high lignin content softwood biomass [2]. Thus, softwood biomass is the most recalcitrant biomass to pretreat for enzymatic hydrolysis. To utilize the most recalcitrant lignocellulosic materials, an efficient, scalable and cost effective pretreatment method is needed.

Bisulfite pulping was a widely used industrial method for pretreating woody biomass for papermaking industries. In 1867, Benjamin Chew Tilghman invented the use of calcium bisulfite to pulp wood [3,4]. The first commercial sulfite process for sulfite pulp production was built in 1874 in Bergvik, Sweden [5]. Due to its effectiveness in lignin sulfonation and lignin removal from wood, bisulfite or sulfite pulping has been used for almost 14 decades in the papermaking industry. In addition, spent sulfite pulping liquor is rich in hemicellulose sugars, such as arabinose, galactose, mannose and xylose. However, the spent sulfite liquor also contains some glucose. Spent sulfite liquor has been a source of hemicellulosic sugar for renewable biofuel fermentation, as demonstrated by Tembec for over two decades [6].

Sulfite pulping sludge has been found highly digestible by enzymes and suitable for producing lignocellulosic ethanol [7-9]. The cost that goes into producing a bleached sulfite pulp, however, is high compared to the price of the sugar that it could replace. Conventional bisulfite pulping time is as long as 6–10 hours [10], and the calcium bisulfite usage is about 31–35% on wood, equivalent to 9.7–10.9 total combined SO_2_ on wood [11]. Zhu et al. [12] introduced a sulfite pretreatment to overcome recalcitrance of lignocellulose (SPORL) where the effective pretreatment was conducted at a higher temperature of 180°C for 30 minutes with 8–10% bisulfite and 1.8–3.7% sulfuric acid on wood. Faster pretreatment time, a bisulfite charge much less than that of bleachable grade bisulfite pulping, and avoiding bleaching and related unit operations downstream makes the cost of pretreatment more consistent with what can be a cost-effective replacement for sugar.

In this paper, we introduce CLE Sugar, which begins with a mild bisulfite pretreatment and results in a high yield pretreated feedstock that enables enzymatic hydrolysis of even recalcitrant feedstocks such as softwood. It is operated at time and temperature conditions intermediate between SPORL and conventional bisulfite pulping times, uses less than half the bisulfite chemical as bisulfite pulping, and avoids the addition of any other acid. Thus, the mild bisulfite step lowers the cost of producing highly enzymatically digestible biomass and for subsequent lignocellulosic sugar production.

## Results

### Biomass feedstocks

The carbohydrate compositions of softwood chips (un-debarked Loblolly pine chips), hardwood chips (debarked maple chips) and switchgrass (Alamo variety) were analyzed. Table 1 below summarizes the carbohydrate composition of the biomass used. The carbohydrate composition of each biomass was determined by converting the polymeric sugars in the feedstock into monomeric sugars such as glucose, xylose, mannose, arabinose and galactose. Results in Table 1 show the original polymeric sugar composition of the biomass. The total polymeric sugar composition for un-debarked Loblolly pine chips, debarked maple chips and switchgrass samples used in the current study was 51.8%, 58.6% and 59.5%, respectively.

**Table 1:** Biomass carbohydrate composition

**Polymer sugar on biomass**	**Softwood un-debarked loblolly pine chips (21A)**	**Hardwood debarked maple chips (22A)**	**Switchgrass Alamo variety (25A)**
Arabinan (%)	1.89	0.51	3.30
Galactan (%)	3.01	0.47	1.04
Glucan (%)	33.2	41.1	33.2
Xylan (%)	5.90	14.3	21.7
Mannan (%)	7.81	2.17	0.22
Total (%)	51.8	58.6	59.5

The wood chips were re-chipped with a Bearcat garden chipper with a 1.91 cm screen to obtain smaller size chips. 3-mm round-hole fines were removed to avoid circulation problems in a 28.3-liter pilot pretreatment reactor. The resulting wood chip size distribution for the softwood chips was 24% “pin size” chips (passes a 7 mm round hole screen, retained on a 3 mm round hole screen), and 76% “accept size” chips (retained on a 7 mm round hole screen). All softwood chips were less than 8 mm thickness. The lengths of the re-chipped chips ranged approximately from 20 to 40 mm.

### Pretreatment

A few biomass feedstocks, including un-debarked Loblolly pine chips, debarked maple chips, and Alamo switchgrass were pretreated in the pilot digester with the mild bisulfite pretreatment method as described in the method section. Table 2 shows the pretreated biomass yield and prehydrolysate amount after the pretreatment. Due to the mild bisulfite pretreatment condition, a high biomass yield was obtained at 74.9%, 69.3%, and 81.6%, respectively for un-debarked Loblolly pine chips, debarked maple chips, and switchgrass. The prehydrolysate has a reducing sugar titer around 2.6–3.7% that is mostly hemicellulose sugars. The prehydrolysate sugars account for 15, 18, and 11% of the biomass sugar, respectively for un-debarked Loblolly pine chips, debarked maple chips and switchgrass. In the pretreatment chemistry and mild conditions, the furan compound formation was 1.29% of biomass feedstock for Loblolly pine and 1.10% for maple, as shown in Table 3.

**Table 2:** Biomass parameters before and after acidic calcium bisulfite pretreatment

**Biomass and yields**	**Softwood un-debarked loblolly pine chips (21A: 165°C, 75 min)**	**Hardwood debarked maple chips (22A: 155°C, 75 min)**	**Switchgrass Alamo variety (25A: 155°C, 75 min)**
Loaded biomass (kg, OD)	3.00	3.00	2.17
Yield (% wt)	74.89	69.25	81.61
Yielded biomass (kg, OD)	2.25	2.08	1.64
Prehydrolysate pH	1.15	1.41	1.56
Prehydrolysate (liter)	9.90	9.43	6.07
Prehydrolysate sugar titer (%)	2.62	3.69	2.30

**Table 3:** Furan formation

**Furan yield on biomass**	**Softwood un-debarked loblolly pine chips (21A)**	**Hardwood debarked maple chips (22A)**	**Switchgrass Alamo variety (25A)**
Hydroxymethylfurfural (%)	0.40	0.25	0.00
Furfural (%)	0.89	0.85	0.22
Total Furan on wood (%)	1.29	1.10	0.22

The pretreated biomass carbohydrate compositions are shown in Table 4. Table 2 shows the hemicellulose solubilization and hydrolysis to hemicellulosic sugars in the prehydrolysate. These hemicellulosic sugars include arabinose, galactose, xylose and mannose. The hemicellulose glucomannan also contains glucose, which was present in small amount in the prehydrolysate due to the glucomannan hydrolysis to glucose and mannose. The pretreated biomass solid has a higher glucan composition and lower hemicellulose composition. The higher glucan composition was also due to the partial dissolution of sulfonated lignin into the prehydrolysate (data not shown).

**Table 4:** Pretreated biomass carbohydrate compositions

**Polymer sugar on pretreated biomass**	**Softwood un-debarked loblolly pine chips (21A)**	**Hardwood debarked maple chips (22A)**	**Switchgrass Alamo variety (25A)**
Arabinan (%)	0.35	0.16	0.79
Galactan (%)	0.78	0.22	0.52
Glucan (%)	40.60	54.9	39.9
Xylan (%)	1.68	7.06	9.78
Mannan (%)	2.84	1.12	0.31
Total (%)	46.3	59.45	51.3

### Enzymatic hydrolysis and total sugar yield

High dosage enzymatic hydrolysis is used to assess the maximum amount of sugar that can be enzymatically released from the pretreated biomass. A low consistency hydrolysis of 5% pretreated biomass was used. Each gram of pretreated biomass was applied with a high enzyme dose of 0.34 g Sigma enzyme mixture. The total sugar yield is defined as the total monomeric sugars from the prehydrolysate and from the enzymatic hydrolysis of the pretreated biomass. The total sugar yields are summarized in Table 5. The results indicate that the total sugar yields from the pretreatment were 85.8%, 94.0% and 80.2%, respectively for un-debarked Loblolly pine chips, debarked maple chips and switchgrass.

**Table 5:** Sugar yields from pretreated biomass

**Sugar and yields on monomeric sugar basis**	**Softwood un-debarked loblolly pine chips (21A)**	**Hardwood debarked maple chips (22A)**	**Switchgrass Alamo variety (25A)**
Biomass amount (kg)	3.00	3.00	2.17
Sugar from biomass (kg)	1.73	1.96	1.24
Sugar in prehydrolysate (kg)	0.26	0.35	0.14
Sugar from hydrolysate (kg)	1.23	1.49	0.85
Total sugar yields (%)	85.8	94.0	80.2

Higher solids hydrolysis is required to achieve a high sugar titer. Table 6 summarizes the total sugar titer of enzymatic hydrolyses at 5% and 17% solid consistency for the pretreated un-debarked Loblolly pine chips. At 17% solids hydrolysis, a lower enzyme dose of 0.075 g enzyme mixture per gram of pretreated biomass was used. This translates into an enzyme dose of 0.056 g enzyme mixture per gram of untreated biomass. With this low enzyme dosage, the total sugar yield was 76.6% for the un-debarked Loblolly pine chips. In addition, Novozymes CTec2 enzyme product was also tested on the high solids loading with an enzyme dosage of 0.044 g CTec2 product per gram of pretreated biomass or 0.033 g CTec2/g untreated wood. CTec2 achieved a total sugar yield of about 85%. This indicated that the CTec2 enzyme product has a higher specific activity than the Sigma enzyme mixture.

**Table 6:** Sugar titer from low and high consistency hydrolysis of pretreated un-debarked Loblolly pine chips

**Test no.**	**Pretreated biomass load (% wt/vol)**	**Enzyme dosage (g/g solid)**	**Hydrolysis time (day)**	**Sugar titers (% wt)**	**Total sugar yield (%)**
1 (Sigma Enzyme Mixture)	5.0	0.342	3	2.7	85.8
2 (Sigma Enzyme Mixture)	17.0	0.075	4	8.1	76.6
3. (CTec2 Enzyme)	17.0	0.044	4	9.2	85.0

Note: Sugar titers and yields were averaged from duplicate experiments.

## Discussion

The mild bisulfite pretreatment provided high yield in both pretreatment and enzymatic hydrolysis with lower furan formation. The CLE Sugar process, while as yet unoptimized, is effective on un-debarked Loblolly pine chips, debarked maple chips, and switchgrass. The total pretreatment time is 1.5 to 2.25 hours including temperature ramp-up time, which is much shorter than 6–10 hours in the conventional sulfite pulping process. The mild bisulfite step provides much higher biomass throughput and uses much less chemical on biomass than conventional sulfite pulping. It also does not require bleaching, a significant cost in conventional sulfite pulping. In fact, the pretreated biomass can be enzymatically hydrolyzed even without washing, resulting in a simpler process. Washing may prove beneficial, however.

**Figure 1:**
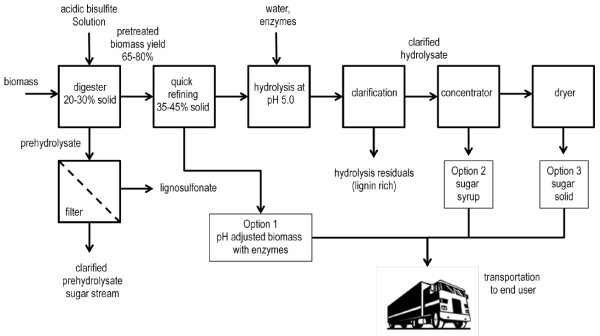
Lignocellulosic sugar production options from lignocellulosic biomass pretreated in a mild bisulfite process.

The pretreated biomass is highly digestible by enzymes and thus can provide a few process options for lignocellulosic sugar production and its sugar integration in a lignocellulosic biofuel or renewable chemical plant. These options include (1) pretreated biomass materials preloaded with enzymes, (2) lignocellulosic sugar syrup and (3) lignocellulosic sugar solids. A process flow chart is shown in Figure 1 for the three lignocellulosic sugar options.

(1). *Pressed pretreated materials without and with enzyme*: After the pretreatment, the pretreated biomass is refined without water addition. The lignosulfonate is removed as a byproduct from the prehydrolysate. The lignosulfonate less prehydrolysate is adjusted to a pH that can neutralize the pretreated biomass to pH 5.0. The pH adjusted material is pressed to a solid density, e.g. 40% or higher. After pH adjustment and pressing, the high density pretreated biomass can be sprayed with sufficient amount of enzyme and properly wrapped under clean-in-place (CIP) condition. This pressed biomass loaded with enzymes can be shipped from a centralized biomass pretreatment center to existing sugar users, such as ethanol plants, for hydrolysis and conversion. This option requires the end user to install a hydrolysis tank for hydrolysate production before a conversion process, but a benefit of shipping the pretreated biomass with enzymes is that initial hydrolysis takes places faster, reducing the amount of time required to liquefy the pretreated feedstock. In some applications, simultaneous saccharification fermentation can be applied depending on end user’s process specifications.

(2). *Lignocellulosic sugar syrup*: To ease the end user’s application in utilizing pretreated biomass, lignocellulosic sugar syrup can be first produced in the centralized lignocellulosic sugar production plant. The pretreated biomass is first enzymatically hydrolyzed to a hydrolysate, which is clarified to removal insoluble solids. The clarified lignocellulosic sugar hydrolysate can be concentrated to lignocellulosic sugar syrup that has a total sugar titer of 50–70%. Due to the high density and sugar titer of lignocellulosic sugar syrup, transportation cost of sugar syrup to a biofuel plant will be less than shipping the untreated biomass or the pretreated biomass. The use of lignocellulosic sugar syrup is much easier than the pretreated biomass since the lignocellulosic sugar stream can be easily blended into the existing process.

(3). *Lignocellulosic sugar solid*: Another option of lignocellulosic sugar format is the solid lignocellulosic hydrolysate. The lignocellulosic sugar syrup can be further processed to evaporate most of the water content to form a lignocellulosic sugar solid. This sugar solid has over 80-90% sugar content and can be bagged for shipping to the end user for a biofuel or biochemical production process that requires a much higher concentration of sugar in their feed stream. The sugar solid has minimal moisture content and properly sealed sugar solids can be stored without requiring a tank.

Lignosulfonate in the liquor stream can be sold as a co-product, or could be concentrated, dewatered and burned along with other solid residuals rich in lignin from the hydrolysis process for energy production as part of power supply for the pretreatment facility. At the yields investigated, the majority of the lignin remains in the solid residuals.

## Conclusions

The CLE Sugar process can supply concentrated lignocellulosic sugars from recalcitrant biomass sources to existing sugar users. Mild bisulfite pretreatment is an efficient step for pretreating recalcitrant biomass to achieve a high yield of total biomass carbohydrate conversion to monomeric lignocellulosic sugars. The historical sulfite pulp industry provides a proven commercial scale of this type of pretreatment technology. Furthermore, the process is flexible for various biomass feedstocks, including softwood (un-debarked Loblolly pine chips), hardwood (debarked maple chips), and herbaceous biomass (Alamo variety switchgrass).

Options for using lignocellulosic sugar produced from the CLE Sugar process include (1) pretreated biomass with enzyme addition, (2) lignocellulosic sugar syrup, and (3) lignocellulosic sugar solid. These options provide a unique advantage of using lignocellulosic sugar in existing production infrastructure. For example, the use of lignocellulosic sugar can help a corn or grain-based ethanol plant by supplementing its feedstock to increase the value of the ethanol by making use of the Renewable Fuel Standard incentives or to maintain feedstock options in the face of variations in corn prices.

## Methods

### Biomass feedstocks

The softwood feedstock was forest chips of Loblolly pine, so they contained bark. Hardwood chips used in this test were debarked maple chips from Michigan. Both the un-debarked Loblolly pine chips and debarked maple chips were re-chipped with a Bearcat garden chipper with a 1.91 cm screen to obtain smaller chips, and 3-mm round-hole fines were removed. Switchgrass biomass is Alamo variety harvested in winter from a Catchlight Energy field trial in the southeastern United States.

### Acidic calcium bisulfite reagent

An acidic calcium bisulfite solution was used as a sulfonation reagent in the pretreatment of biomass. Calcium bisulfite was produced by constantly purging pure sulfur dioxide into a calcium oxide solution. The final calcium bisulfite concentration contained about 2–4% total sulfur dioxide, of which about 1% was free sulfur dioxide. The pH of this calcium bisulfite solution was about 1.4.

The total sulfur in the reagent was determined by a standard ASTM D 1552–90 method of sulfur in petroleum products (high temperature method) in Leco S632 Sulfur Determinator (St. Joseph, Michigan). The sulfur result was used to calculate the total equivalent sulfur dioxide (bound and free). The calcium in the liquor was measured by a standard method equivalent to EPA Method 200.8, revision 5.4, of EPA/600/R-94/111. The calcium in the liquor was analyzed by an inductively coupled argon plasma mass spectrometry (ICPMS) (Thermo Elemental X Series II, Waltham, MA). The bound sulfur dioxide in the calcium bisulfite was determined by the total amount of soluble calcium in the liquor, and the free sulfur dioxide was determined by subtracting the bound sulfur dioxide from the total sulfur dioxide.

### Pilot pretreatment digester

A 28.3-liter (one cubic foot) digester with a heat exchange was used in biomass pretreatment with an acidic calcium bisulfite reagent. The digester and the heat exchanger are made of SA-515 Gr 70 alloy and the digester head is made of SA-515 Gr C alloy. The piping for calcium bisulfite reagent circulation during the pretreatment is made up of 304 stainless. During pretreatment, steam indirectly heats up the cooking liquor that circulates back to the digester.

### Sulfonation conditions

The re-chipped biomass materials were sulfonated in the pilot pretreatment reactor. In each cook, 3.0 kilograms (oven dried - OD basis) of woody biomass material were used at a biomass-to-liquor ratio of about 4.0, and 2.2 kilograms (oven dried basis) of switchgrass was used at a biomass-to-liquor ratio of about 4.5.

The biomass materials were first steamed to 90°C to remove air in the void space of the biomass. After the condensation was drained, the woody biomass was charged with a cool liquor of calcium bisulfite at 12.5% on wood. The pre-steamed un-debarked Loblolly chips were heated up to 165°C in 15 minutes and held at 165°C for 75 minutes. The pre-steamed debarked maple chips were heated up to 155°C in 15 minutes and held at 155°C for 75 to 120 minutes. Similarly, the pre-steamed switchgrass was heated up to 155°C in 15 minutes and held at 155°C for 75 minutes. The wet cooked biomass and the prehydrolysate amounts were measured for mass balance calculation. After cooking, a prehydrolysate “spent liquor” fraction was drained and the cooked chips were collected after having relieved the pressure in the pretreatment reactor by draining the spent liquor and venting. The cooked biomass solid content was measured by drying a biomass sample of about 70 wet grams in an oven set at 105°C for overnight. This solids content was used to determine the total recovered solid biomass after the pretreatment.

The cooked chips were very mushy. The cooked wood chips were passed once through an Alpine grinder, without adding any water, to form a pulp-like material. The cooked switchgrass was not refined due to its pulpy status upon removal from the pretreatment reactor.

### Biomass Analysis Methods

The carbohydrate analysis in biomass feedstocks and in pretreated biomass samples was determined by hydrolyzing 100 mg of refined materials with 72% sulfuric acid at 127°C for 60 minutes for a complete carbohydrate hydrolysis. The monomeric sugars from completely acid-hydrolyzed biomass were analyzed quantitatively in the dilute sample for glucose, xylose, mannose, arabinose and galactose in a Dionex ion exchange chromatography. The Dionex chromatography is equipped with a CarboPac PA1 (Dionex P/N 035391) 4 mm × 250 mm ion-exchange column and a Dionex ED 40 pulsed amperometric detector with gold working electrode and solid state reference electrode. The monomeric sugar results were used for the carbohydrate composition calculation in a biomass sample or a pretreated biomass sample.

All biomass carbohydrates were completed in single analysis with duplicate tests of a known composition control (e.g. debarked and clean Southern Loblolly pine chip sample) to assure the accuracy of the analysis in Weyerhaeuser’s analytical group.

In this study, lignin, uronic acids, acetyl groups, extractives and ash were not quantitatively studied.

### Enzymatic hydrolysis

For enzymatic hydrolysis, a pre-mixed enzyme cocktail was formulated with a cellulase product (Sigma Cat. No. C2730) at 99.5 mg protein/mL, a beta-glucosidase product (Sigma Cat. No. C6105) at 42.5 mg protein/mL, and a xylanase (Sigma Cat. No. X2753) at 3.4 mg protein/mL. The total mixed Sigma enzyme protein titer was 145.5 mg/mL. The Sigma enzyme cocktail has a density of 1.1 g/ml. Cellic® CTec2 enzyme product was provided by Novozymes and was used in the high consistency pretreated biomass hydrolysis. The CTec2 enzyme product has a density of 1.2 g/ml. In the enzymatic hydrolysis, the enzyme dosage is defined as gram of enzyme product per oven dry (OD) gram of pretreated biomass. Unwashed pretreated biomass was used in all the enzymatic hydrolysis tests. The enzymatic hydrolysis was conducted in screw-capped 50-mL volume in 125 mL Erlenmeyer flasks in an orbital shaking incubator, controlled at 50°C and 200 r.p.m. The hydrolysis pH was controlled at pH 4.8 by a 50 mmol sodium citrate buffer.

### Sugar analysis

One milliliter hydrolysis sample was weighed and diluted into a total volume of 10 mL in deionized water. The sample was then centrifuged and the supernatant was used for sugar analysis in a HPLC. A Shimadzu HPLC equipped with a 300 × 7.8 mm Bio-Rad Aminex HPX-87P Column (Cat. No. 125–0098) was used to analyze glucose, xylose, mannose, arabinose and galactose in the hydrolysate from an enzymatic hydrolysis and in the prehydrolysate from a cook. The 87P column was run with water as an eluent at 0.6 ml/min at 85°C. The acetic acid in the prehydrolysate was analyzed in a 300 × 7.8 mm Bio-Rad Aminex HPX-87H Column (Cat. No. 125–0140) with 0.005M sulfuric acid as an eluent at 0.6 ml/min at 65°C. Furfural and hydroxymethylfurfural were analyzed in either the HPX-87P or the HPX-87H column.

## Abbreviations

CLE: Catchlight Energy;CIP: Clean-in-place;SPORL: Sulfite pretreatment to overcome recalcitrance of lignocellulose

## Competing interests

The authors have also authored patent applications for Catchlight Energy that are relevant to this subject matter.

## Authors’ contributions

JG designed the experiments and drafted this manuscript. DA conceived of process options for delivering cellulosic biomass to existing assets. BL assisted with the design of experiments. All authors read and approved the final manuscript.

## Authors’ information

Dr. Johnway Gao is Senior R&D Engineer, Dr. Dwight Anderson is Bioconversion Manager, and Dr. Benjamin Levie is Senior Process Engineer with Catchlight Energy and are based in Federal Way, Washington.
